# PPT1 inhibition enhances the antitumor activity of anti–PD-1 antibody in melanoma

**DOI:** 10.1172/jci.insight.133225

**Published:** 2020-09-03

**Authors:** Gaurav Sharma, Rani Ojha, Estela Noguera-Ortega, Vito W. Rebecca, John Attanasio, Shujing Liu, Shengfu Piao, Jennifer J. Lee, Michael C. Nicastri, Sandra L. Harper, Amruta Ronghe, Vaibhav Jain, Jeffrey D. Winkler, David W. Speicher, Jerome Mastio, Phyllis A. Gimotty, Xiaowei Xu, E. John Wherry, Dmitry I. Gabrilovich, Ravi K. Amaravadi

**Affiliations:** 1 Abramson Cancer Center and Department of Medicine,; 2 Department of Systems Pharmacology and Translational Therapeutics and Penn Institute for Immunology, and; 3 Department of Pathology and Laboratory Medicine, Perelman School of Medicine, University of Pennsylvania, Philadelphia, Pennsylvania, USA.; 4 Department of Chemistry, College of Arts & Sciences, University of Pennsylvania, Philadelphia, Pennsylvania, USA.; 5 Wistar Institute, Philadelphia, Pennsylvania, USA.; 6 Department of Biostatistics, Epidemiology & Informatics, Perelman School of Medicine, University of Pennsylvania, Philadelphia, Pennsylvania, USA.

**Keywords:** Oncology, Therapeutics, Autophagy, Lysosomes, Melanoma

## Abstract

New strategies are needed to enhance the efficacy of anti–programmed cell death protein antibody (anti–PD-1 Ab) in cancer. Here, we report that inhibiting palmitoyl-protein thioesterase 1 (PPT1), a target of chloroquine derivatives like hydroxychloroquine (HCQ), enhances the antitumor efficacy of anti–PD-1 Ab in melanoma. The combination resulted in tumor growth impairment and improved survival in mouse models. Genetic suppression of core autophagy genes, but not *Ppt1*, in cancer cells reduced priming and cytotoxic capacity of primed T cells. Exposure of antigen-primed T cells to macrophage-conditioned medium derived from macrophages treated with PPT1 inhibitors enhanced melanoma-specific killing. Genetic or chemical Ppt1 inhibition resulted in M2 to M1 phenotype switching in macrophages. The combination was associated with a reduction in myeloid-derived suppressor cells in the tumor. Ppt1 inhibition by HCQ, or DC661, induced cyclic GMP-AMP synthase/stimulator of interferon genes/TANK binding kinase 1 pathway activation and the secretion of interferon-β in macrophages, the latter being a key component for augmented T cell–mediated cytotoxicity. Genetic *Ppt1* inhibition produced similar findings. These data provide the rationale for this combination in melanoma clinical trials and further investigation in other cancers.

## Abstract 2

Inhibiting palmitoyl-protein thioesterase 1 (PPT1), a target of CQ derivatives like hydroxychloroquine (HCQ), enhances the antitumor efficacy of anti-PD-1 Ab in murine melanoma models.

## Graphical abstract

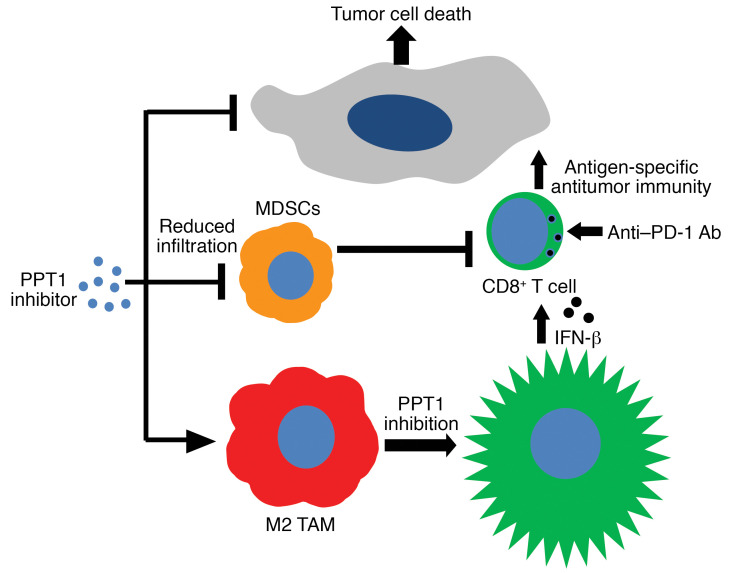

## Introduction

While there have been extensive efforts to combine other T cell–stimulating factors with anti–programmed cell death protein antibody (anti–PD-1 Ab), there is an increasing interest in identifying T cell–independent strategies that will augment the efficacy of anti–PD-1 Ab. Tumor cell autophagy has been identified as a major resistance mechanism to targeted therapy and chemotherapy. Chloroquine and hydroxychloroquine (CQ and HCQ) are the only drugs that have been tested as autophagy inhibitors in clinical trials in patients with cancer. However, there are conflicting reports about whether as a single agent CQ derivatives augment, impair, or have no effects on antitumor immunity (1, 2).

Recently, we identified the major molecular target of CQ derivatives as the lysosomal protein palmitoyl-protein thioesterase 1 (PPT1) (3, 4). Meanwhile, multiple clinical trials involving HCQ in combination with chemotherapy or targeted therapy have demonstrated the safety of HCQ combination regimens and some preliminary antitumor activity in patients (5, 6). To date, the role of PPT1 in cancer immunotherapy has remained unexplored. Therefore, we conducted preclinical studies with the clinically used PPT1 inhibitor HCQ in 2 immunocompetent mouse melanoma models and demonstrated an enhancement of tumor response when anti–PD-1 Ab was combined with HCQ. In vitro, HCQ, D661 (a more potent PPT1 inhibitor), or small interfering RNA against *Ppt1* (si*Ppt1*) were all able to convert M2 to M1 tumor-associated macrophages (TAMs). Conditioned media from macrophages treated with Ppt1 inhibitors were able to enhance T cell–mediated cancer cell killing. Interestingly, the combination of HCQ and anti–PD-1 Ab resulted in a change in TAM polarization and a significant reduction in myeloid-derived suppressor cell (MDSC) infiltration in vivo while each single agent did not. The mechanism by which PPT1 inhibitors produced macrophage phenotype switching was dependent on mitochondrial calcium release and p38 activation. PPT1 inhibition also induced the cyclic GMP-AMP synthase/stimulator of interferon genes/TANK binding kinase 1 (cGAS/STING/TBK1) pathway to induce IFN-β release from macrophages. PPt1 inhibition–induced IFN-β release was critical for the augmentation of antigen-primed T cell killing of melanoma cells. These data suggest that this combination, which can be immediately tested in the clinic, could provide an alternative rational combination approach for melanoma immunotherapy.

## Results

**Figure 1:**
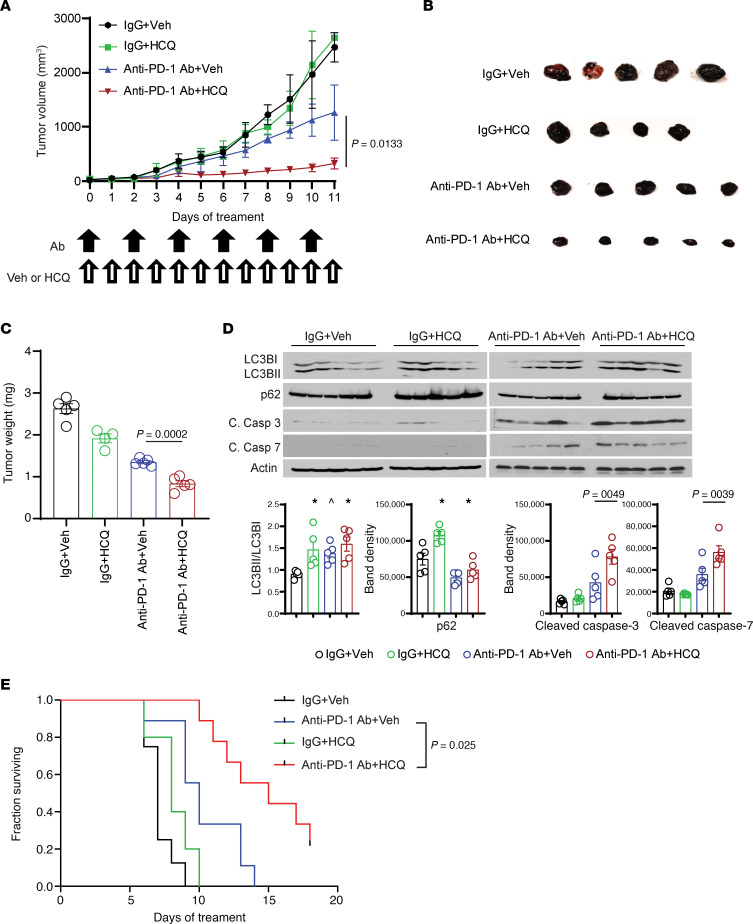
PPT1 inhibitor HCQ enhanced the antitumor efficacy of anti–PD-1 Ab in a mouse melanoma model. (**A**) Tumor growth curve: 5 × 10^5^ B16 cells were injected in the flank of C57BL6/J mice. After tumors reached the size of 50 mm^3^, mice were randomized to cohorts of *n* = 5 mice each. Mice were treated with either IgG control 200 μg or anti–PD-1 Ab 200 μg every other day and either water or HCQ 60 mg/kg i.p. every day, and tumors were measured every day. (**B**) Picture of excised B16 tumors. (**C**) Final tumor weight. (**D**) Immunoblot analysis of autophagy (LC3B-I/II, SQSTM1/p62) and apoptosis markers (caspase-3 and caspase-7) with quantification of the bands. One-way ANOVA and Dunnett’s procedure. (**E**) Kaplan-Meier survival curve of mice treated as above in a new experiment. Exact log-rank test. The average with SEM was calculated for each treatment cohort. A *P* value is presented for the 2-tailed *t* test of the hypothesis that the addition of HCQ to anti–PD-1 Ab is significantly different compared with anti–PD-1 Ab + Veh; * indicates adjusted *P* < 0.05, and ^ indicates adjusted *P* < 0.10 testing the hypothesis that the mean relative risk is different from 1.

In order to determine if autophagy inhibition could augment the efficacy of immunotherapy, we treated B16 melanoma tumors with IgG, HCQ, anti–PD-1 Ab, and anti–PD-1 Ab and HCQ. Only the combination of anti–PD-1 Ab and HCQ significantly impaired tumor growth (Figure 1A) without any sign of toxicity (Supplemental Figure 1A; supplemental material available online with this article; https://doi.org/10.1172/jci.insight.133225DS1). Tumors harvested at the end of the experiment were significantly smaller with combination compared with monotherapy arms (Figure 1, B and C). Unlike a previous report (7), we found no change in mTOR signaling or autophagy induction in B16 melanoma cells treated with anti–PD-1 Ab (data not shown), and anti–PD-1 Ab + HCQ did not show any cytotoxicity in B16 melanoma cells in vitro (Supplemental Figure 1B). In B16 tumors, however, the LC3II/I ratio was increased and p62 level was reduced albeit to a modest degree with anti–PD-1 Ab, reflecting the induction of autophagy in vivo (Figure 1D). Accumulation of autophagic vesicles was observed in electron micrographs of a tumor biopsy of a melanoma patient treated with pembrolizumab (Supplemental Figure 1C). Anti–PD-1 Ab and HCQ produced significantly increased apoptosis in tumor tissue (Figure 1D) and increased the survival of mice compared with anti–PD-1 Ab alone (Figure 1E).

**Figure 2:**
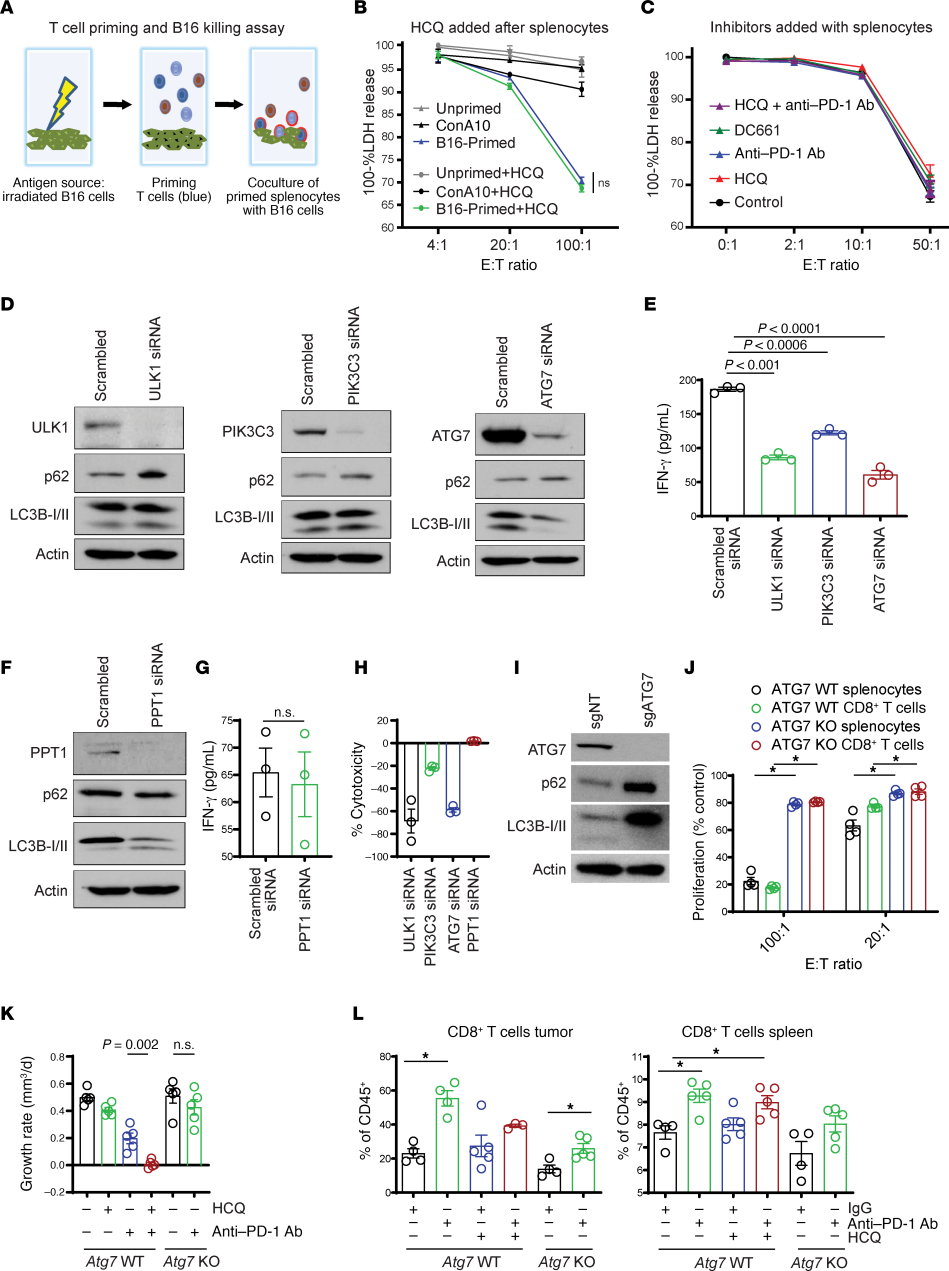
The effects of PPT1 inhibition are not recapitulated by *Ulk1*, *Pik3c3*, and *Atg7* KD or *Atg7* KO, and HCQ does not impair CD8^+^ T cell–mediated killing. (**A**) Schematic of priming and coculture experiments to measure antigen-specific T cell killing in vitro. (**B**) One hundred percent lactate dehydrogenase (LDH) from the coculture of primed or unprimed splenocytes with live B16, in the presence or absence of HCQ (10 μM). Each experiment was performed in triplicates, and results were reproduced with 3 independent experiments. Concanavalin A was used as a nonspecific splenocyte priming agent. (**C**) One hundred percent LDH measurement in primed splenocytes cocultured with B16 with/without indicated treatments. (**D**) Immunoblots confirming the B16 *Ulk1*-, *Pik3c3*-, and *Atg7*-KD status. (**E**) Dot plot representing ELISA performed for the measurement of splenocyte-secreted IFN-γ upon coculturing with B16 with *Ulk1*-, *Pik3c3*-, and *Atg7*-KD conditions. Each experiment was performed in triplicates, and the results were reproduced by 3 independent experiments. (**F**) Immunoblot confirming the B16 *Ppt1*-KD status. (**G**) Dot plot representing IFN-γ ELISA in *Ppt1*-KD B16 coculture as above in **E** for 3 independent experiments. (**H**) Measurement of percentage T cell–mediated cytotoxicity of B16 cells in *Ulk1*, *Pik3c3*, *Atg7*, and *Ppt1* condition. (**I**) Immunoblot confirming the B16 *Atg7*-KO status. (**J**) Irradiated B16-primed splenocytes or purified splenic CD8^+^ T cells were cocultured with B16 WT *Atg7* or B16 *Atg7*-KO cells, and the percentage proliferation was measured by performing 3 independent experiments. (**K**) B16 Cas9 control or B16 *Atg7*-KO cells (5 × 10^5^) were injected into the flanks of C57BL6/J mice. After tumors reached a size of 50 mm^3^, mice were randomized to cohorts of *n* = 5 mice each, and B16 Cas9 control tumors were treated with IgG control + vehicle, anti–PD-1 Ab (200 μg i.p. every other day) + vehicle, IgG + HCQ (60 mg/kg i.p. daily), or the combination. B16 *Atg7*-KO tumors were treated with either IgG control or anti–PD-1 Ab. The average growth rate ± SEM is shown. (**L**) Tumors and spleens were harvested from the experiment in **B**, on day 8 of treatment. The percentage of CD8^+^ T cells in CD45^+^ cells in spleen and tumor are shown. All *t* tests were 2 tailed (**G** and **K**). One-way ANOVA and Bonferroni’s adjustment (**B**, **C**, and **J**) or Dunnett’s procedure (**E** and **L**). E:T, effector/target; sgNT, single guide nontargeting.

To understand the effects of autophagy inhibition on T cell priming or effector T cell function, we performed an in vitro priming and coculture experiment using either C57BL6/J splenocytes or purified CD8^+^ T cells isolated from spleens. For priming we exposed splenocytes or CD8^+^ T cells to irradiated B16 cells. Next these primed splenocytes or CD8^+^ T cells were cultured with live B16 cells and cytotoxicity was measured (Figure 2A). Antigen-primed splenocytes or nonspecifically (concanavalin A) activated splenocytes were cocultured in the absence or presence of HCQ with live B16 cells. The addition of HCQ after priming did not impair T cell–mediated killing of tumor cells (Figure 2B). Neither HCQ, the dimeric CQ DC661 (3), nor anti–PD-1 Ab administered before or after priming either augmented or blunted antigen-specific T cell killing of B16 cells (Figure 2C). To study the effects of autophagy inhibition in this system, we systematically knocked down key autophagy genes involved in each of the major autophagy protein complexes that coordinately assemble the autophagosome, including *Ulk1*, *Pik3c3* (Vps34), and *Atg7*. KD of these 3 genes in B16 cells produced reduced autophagic flux as evidenced by increase in p62. While Lc3b-II levels did not change much with si*Ulk1* or si*Pik3c3* as is often observed (8, 9), there was significant loss of Lc3b-II expression as expected with si*Atg7* (Figure 2D). Splenocytes or T cells primed with B16 cells with any of the 3 autophagy genes knocked down produced significantly less IFN-γ stimulation than B16 cells exposed to siNon-target control (Figure 2E). In contrast, KD of *Ppt1*, the lysosomal target of CQ derivatives, in B16 cells resulted in no significant difference in T cell priming, as evidenced by similar IFN-γ secretion compared with siNon-target KD (Figure 2, F and G). KD of *Ulk1*, *Pik3c3*, or *Atg7* in B16 cells during priming resulted in significantly reduced T cell–mediated killing of proliferating B16 cells expressing the same siRNAs. In contrast KD of *Ppt1* did not impair antigen-primed T cells from killing B16 si*Ppt1* cells (Figure 2H). Next, to determine if autophagy induction in tumor cells by anti–PD-1 Ab is a major contributor to the reduced efficacy of anti–PD-1 therapy, CRISPR/Cas9 gene editing was used to knock out an essential autophagy gene *Atg7* in B16 cells (Figure 2I). As was found to be the case with si*Atg7*, B16 *Atg7*-KO cells showed a reduced ability to prime syngeneic splenocytes compared with B16 WT cells (Figure 2J). Tumors were generated in the flanks of C57BL6/J mice using B16 Cas9 WT and B16 *Atg7* KO mouse cell lines. While treatment with anti–PD-1 + HCQ significantly suppressed tumor growth rates compared with monotherapy in B16 *Atg7* WT cells, no such augmentation of anti–PD-1 efficacy was observed in B16 *Atg7*-KO tumors treated with anti–PD-1 (Figure 2K). Immunophenotyping of the tumors described above demonstrated that anti–PD-1 Ab significantly increased the percentage of CD8^+^ T cells in both B16 *Atg7* WT tumors and B16 *Atg7*-KO tumors (Figure 2L). There was no significant difference in the percentage of CD8^+^ T cells in B16 *Atg7* WT tumors from mice treated with anti–PD-1 Ab and HCQ compared with anti–PD-1 Ab alone. In the spleen there were increased CD8^+^ T cells with all treatments given, compared with control in both *Atg7* WT and *Atg7*-KO tumors (Figure 2L). Similarly, there were no significant differences in Ki67^+^ or granzyme B^+^ CD8^+^ T cells in either the tumors or the spleens across all treatments (Supplemental Figure 2). Taken together these findings suggested that the enhanced efficacy of HCQ and anti–PD-1 Ab was not due to a direct effect on T cells nor through autophagy inhibition in tumor cells.

**Figure 3:**
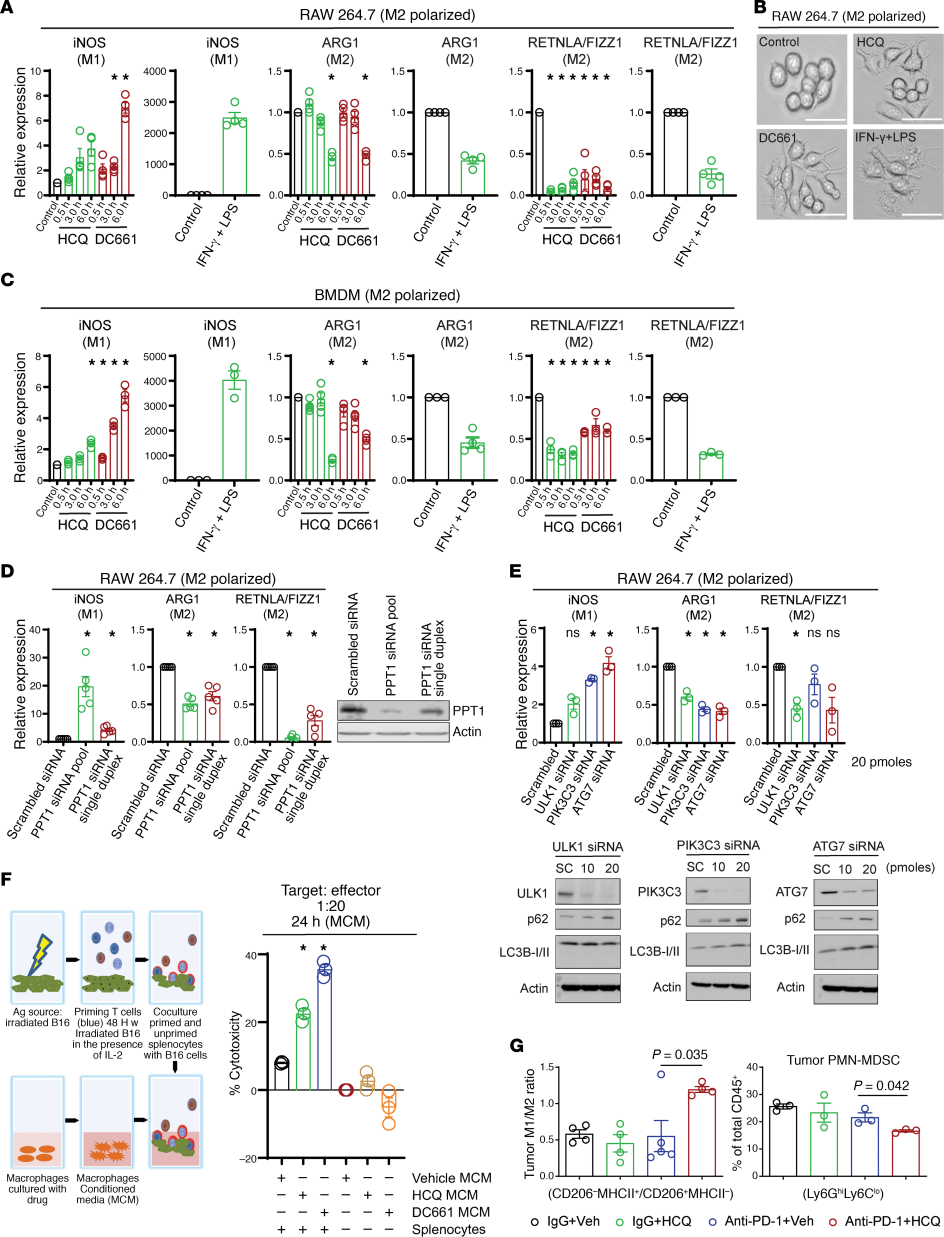
PPT1 inhibition induces a change in macrophage polarization that favors antitumor immunity. (**A**) Dot plot for the quantitative PCR (qPCR) expression in mouse macrophage RAW 264.7 cells polarized to an M2 phenotype following treatment with HCQ 10 μM or DC661 0.6 μM at the indicated time points (in hours) showing the results of 4 independent experiments. (**B**) Bright-field images of RAW 264.7 polarized to an M2 phenotype treated with HCQ or DC661 M1 phenotype control (IFN-γ + LPS). M2 cells have a round morphology whereas drug-treated M2 cells take on an elongated morphology with multiple pseudopodia typical of M1 macrophages (positive control). Images were taken at original magnification ×10. (**C**) Expression of M2 and M1 markers in mouse BMDMs treated with HCQ or DC661 as in **A**. Each result was reproduced by 3 independent experiments. (**D**) Dot plot showing qPCR expression of iNOS and RETNLA/FIZZ1 following *Ppt1* KD in RAW 264.7 cells polarized to an M2 phenotype, and each result was reproduced by 5 independent experiments. Immunoblot showing the KD status of *Ppt1* protein. (**E**) qPCR expression of M1 and M2 markers in *Ulk1*-, *Pik3c3*-, and *Atg7*-KD conditions. Results were reproduced with 3 independent experiments. Immunoblots showing the KD status of *Ulk1*, *Pik3c3*, *Atg7* protein and expression of LC3 and p62. (**F**) Schema of experimental setup and cytotoxicity elicited by primed splenocytes with or without exposure to MCM collected from RAW 264.7 macrophages treated with control HCQ or DC661 for 24 hours. (**G**) Immunophenotyping for M1/M2 ratio of TAMs and percentage PMN-MDSCs in B16 melanoma tumors after 8 days of treatment. Mean and SEM are representative of 4 to 5 replicates, and the experiment was repeated at least 3 times. A *P* value is presented for the test of the hypothesis that the addition of HCQ to anti–PD-1 Ab is significantly different compared with anti–PD-1 Ab + Veh; * indicates an adjusted *P* < 0.05 testing the hypothesis that each experimental group is different from control. All *t* tests were 2 tailed (1 sample in **A** and **C**, 2 sample in **F** and **G**). One-way ANOVA and Dunnett’s procedure (**D** and **E**). Scale bar: 100 μm.

A recent study suggests that single-agent CQ modulates the phenotype of TAMs from a protumorigenic (M2) to an antitumorigenic (M1) phenotype or mouse macrophages in vitro and in vivo (1). To test this, RAW 264.7 mouse macrophages were polarized to an M2 phenotype and treated with HCQ or DC661. Both HCQ and DC661 resulted in M2 to M1 phenotype switching as evidenced by significant changes in inducible nitric oxide synthase (iNOS), arginase 1 (ARG1), and resistin like α/FIZZ1 (RETNLA/FIZZ1) (Figure 3A). Morphologically, there was a striking change in M2 polarized macrophages treated with HCQ or DC661, with cells taking on a spindle shape with increased pseudopodia resembling M1 polarized cells (Figure 3B). These findings were reproduced in mouse bone marrow–derived macrophages (BMDMs) (Figure 3C). *Ppt1* KD also produced a 25-fold increase in iNOS and 2- to 3-fold decrease in RETNLA/FIZZ1, reflecting effective change in polarization from an M2 to M1 phenotype (Figure 3D). KD of core autophagy genes *Ulk1*, *Pik3c3*, and *Atg7* also produced changes in M2 to M1 macrophage polarization (Figure 3E). Ppt1 KD produced 4–6 times the change in iNOS expression compared with KD of other core autophagy genes, suggesting a more efficient macrophage polarization switch was achieved by targeting Ppt1.

CD8^+^ T cells play a major role in the elimination of tumor cells and regression of tumors by their cytotoxic activity (10). To determine if drug-induced changes in macrophage phenotype could contribute in the elimination of tumor cells by T cells, splenocytes were cocultured with irradiated B16 cells and, after priming, cocultured with live B16 cells in the presence or absence of macrophage-conditioned medium (MCM), collected from control, HCQ-treated, or DC661-treated macrophages (Figure 3F). The exposure of HCQ- or DC661-treated MCM to antigen-primed splenocytes significantly enhanced antigen-primed, splenocyte-mediated killing of B16 cells compared with antigen-primed splenocytes exposed to vehicle-treated MCM (Figure 3F and Supplemental Figure 3). Unlike a previous report that showed CQ can change TAM M2 to M1 phenotype in vivo (1), but in concordance with another published report (2) that showed CQ derivatives had no effects on immune infiltrates in the tumor microenvironment, we found no change in immune infiltrates with HCQ or anti–PD-1 alone. However, the combination of anti–PD-1 and HCQ produced a significant 3-fold increase in M1/M2 ratio. The combination also significantly reduced peripheral mononuclear MDSCs (PMN-MDSCs) (Figure 3G), which play a major immunosuppressive role in the tumor microenvironment (11). There were no significant changes in tumor monocytic MDSCs (M-MDSCs), eosinophils, dendritic cells (DCs), CD4^+^ T cells, or CD8^+^ T cells (Supplemental Figure 4, A and B). However, the percentage of NK cells increased significantly with anti–PD-1 Ab and HCQ as compared with IgG control or anti–PD-1 Ab alone (Supplemental Figure 4, B and C). Label-free proteomics of whole B16 tumors demonstrated that proteins related to macrophage biology were significantly increased or decreased with combined anti–PD-1 Ab + HCQ treatment compared with anti–PD-1 Ab alone (Supplemental Figure 4D). These included a 4-fold increase in Rab6a, required for TNF secretion in M1 macrophages (12); a 2-fold increase in Golga7, essential for palmitoylation of proteins (13); a 2-fold decrease in matrix metalloproteinase 12, which stimulates MDSC expansion (14); and a 4-fold decrease in leukemia inhibitory factor, which supports immunosuppressive TAMs (15).

**Figure 4:**
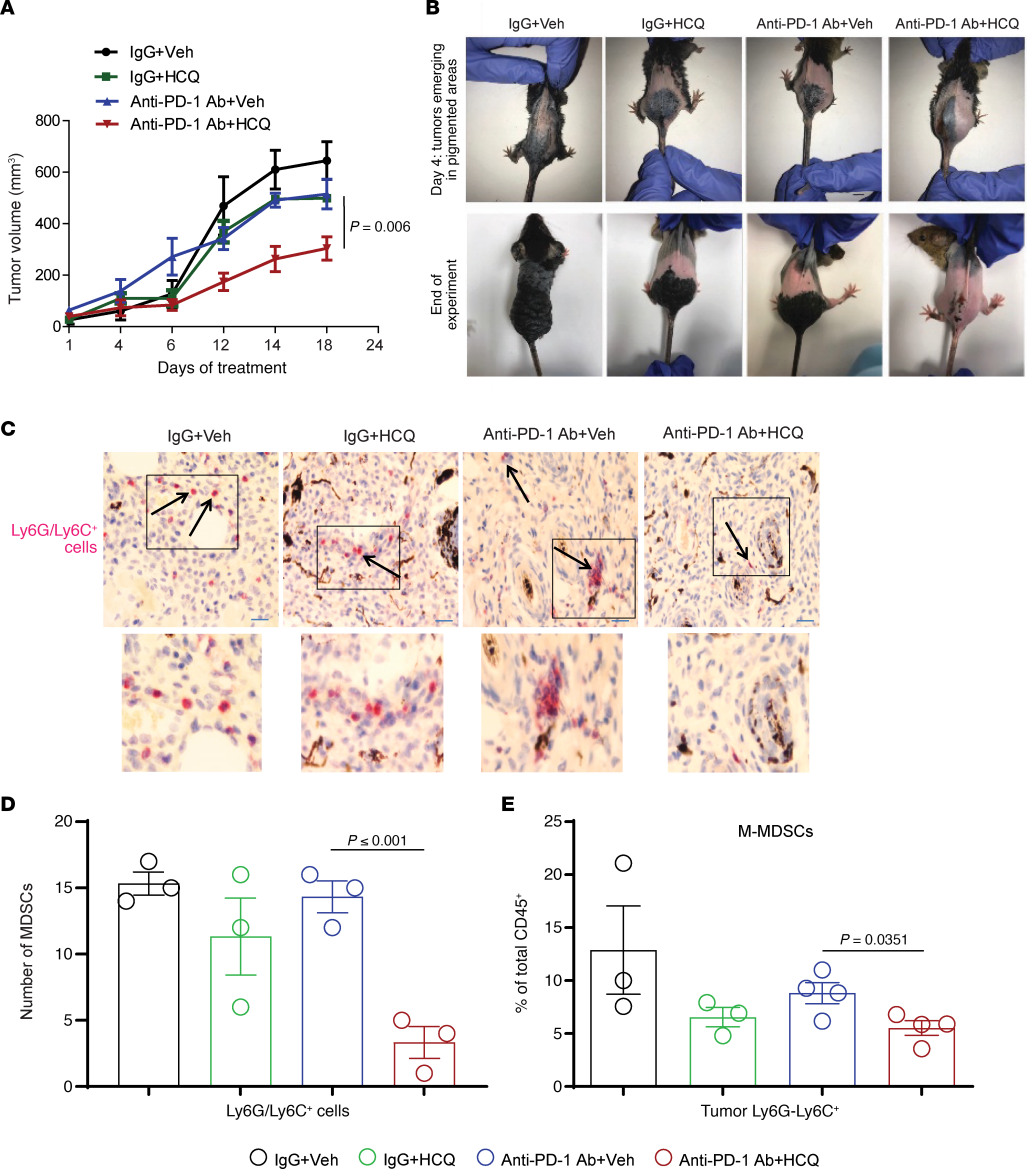
HCQ and anti–PD-1 Ab combination impairs tumor growth and reduces the MDSCs’ infiltration in a *BRaf^CA^ Pten^loxP^ Tyr::CreER^T2^* melanoma model. (**A**) Topical 4-HT was applied on the back to elicit spontaneous melanoma growth (*n* = 4 per treatment), and once tumors were palpable treatment as in Figure 1 was started. (**B**) Representative images of mice. (**C**) Representative images of IHC staining of tumor against Ly6C/Ly6G (MDSC marker) at original magnification ×10 (inset of each image is ×2.5 magnified). (**D**) Number of Ly6C/Ly6G^+^ cells per high-powered field. (**E**) Immunophenotyping of M-MDSCs in tumor. A *P* value is presented for the test of the hypothesis that the addition of HCQ to anti–PD-1 Ab is significantly different compared with anti–PD-1 Ab + Veh. All *t* tests were 2 tailed and 2 sample.

We next used the *BRaf^CA^ Pten^loxP^ Tyr::CreER^T2^* mouse melanoma model. In this model painting the skin of mice with 4 hydroxytamoxifen (4-HT) activates Cre recombinase, and melanocytes express mutant *Braf* and lose *Pten*. Spontaneous melanoma tumors arise on the skin of these mice with 100% penetrance. In concordance with the B16 tumor model, we found that HCQ significantly enhanced the antitumor response of anti–PD-1 Ab (Figure 4, A and B), with no sign of toxicity (data not shown). A significant reduction of Ly6G/Ly6C^+^ MDSCs was also observed with the combination using immunohistochemistry staining of excised tumor tissue (Figure 4, C and D). Interestingly, HCQ with anti–PD-1 Ab reduced the infiltration of M-MDSCs (CD45^+^CD11b^+^Ly6G^lo^Ly6C^hi^) (Figure 4E). There was a significant reduction in tumor CD45^+^CD11b^+^Ly6C^+^Ly6G^–^CX3CR1^+^ monocytes and spleen eosinophils (CD45^+^CD11b^+^Siglec F^+^) and no significant change in DCs (CD45^+^CD11b^+^CD11c^+^) and PMN-MDSCs (CD45^+^CD11b^+^Ly6C^lo^Ly6G^hi^) (Supplemental Figure 5, A and B). There was no significant change in CD8^+^Ki67^+^, CD8^+^Granzyme B^+^, CD4^+^, and Foxp3^+^ (Treg) T cells compartment in the spleen and tumor (Supplemental Figure 6). Taken together these data suggest that CQ derivatives that target PPT1 enhance the antitumor efficacy of anti–PD-1 Ab by targeting both the tumor cell and multiple immunosuppressive myeloid subsets. These effects result in enhancement of T cell–mediated killing without major changes in the T cell populations. Taken together these results show that PPT1 inhibition changes macrophages from an M2 to an M1 polarization state and significantly reduces tumor MDSC infiltration in vivo. It also raises the possibility that PPT1 inhibition stimulates macrophages to secrete a factor that enhances T cell cytotoxicity.

**Figure 5:**
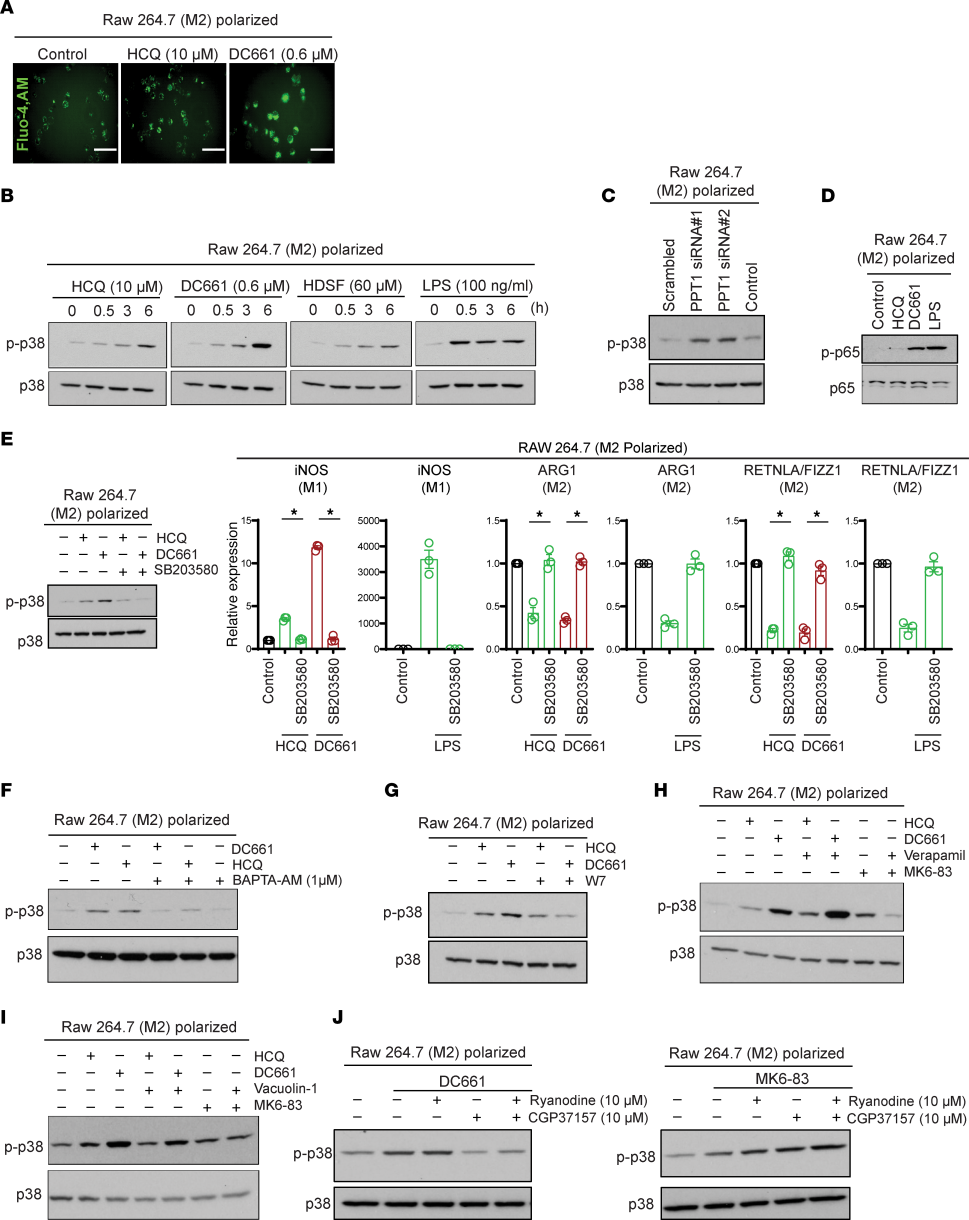
PPT1 acts as a molecular switch, and its inhibition results in calcium-dependent p38 phosphorylation and macrophage polarization. (**A**) Confocal microscopy of RAW 264.7 macrophages for staining calcium by Fluo-4, AM, dye in PPT1 inhibitor HCQ- and DC661-treated cells. Images were taken at original magnification ×40 under Olympus IX71 confocal microscope. (**B**) Immunoblots showing p-p38 and total p38 in DC661-, HCQ-, HDSF-, and LPS-treated RAW 264.7 macrophages. (**C**) Immunoblots for p-p38 and p38 in *Ppt1*-KD condition in RAW 264.7 macrophages. (**D**) Immunoblot representing p-p65 and p65 in HCQ-, DC661-, and LPS-treated macrophages. (**E**) Immunoblots for p-p38 in HCQ or DC661 with cotreatment of p38 inhibitor SB203580 (5 μM) in macrophages. Dot plots representing qPCR expression in mouse macrophage RAW 264.7 cells polarized to an M2 phenotype following treatment with HCQ 10 μM or DC661 0.6 μM or cotreatment with p38 inhibitor as indicated. Each result was reproduced with 3 independent experiments. (**F**) Immunoblots for p-p38 in HCQ and DC661 or cotreatment with calcium chelator BAPTA-AM. (**G**) Immunoblots for p-p38 in HCQ and DC661 or cotreatment with calmodulin inhibitor W7. (**H**) Immunoblots for p-p38 in HCQ, DC661, or TRPML1 agonist MK6-83 or cotreatment with TRPML1 inhibitor verapamil. (**I**) Immunoblots for p-p38 in HCQ, DC661, or TRPML1 agonist MK6-83 or cotreatment with PIKfyve inhibitor vacuolin-1. (**J**) Immunoblot for p-p38 in DC661-treated cells or cotreatment of ER calcium channel inhibitor ryanodine or mitochondrial Na^+^/Ca^2+^ exchanger inhibitor CGP37157. Immunoblot for p-p38 in TRPML1 agonist or cotreatment of ryanodine or CGP37157 in macrophages. Scale bar: 100 μm. * indicates *P* < 0.05. All *t* tests were 2 tailed and 2 sample.

Having established a link between PPT1 inhibition in myeloid cells and T cell killing, we next turned our attention to the mechanism by which CQ derivatives were augmenting antitumor immunity. Previous work showed that CQ activates NF-κB signaling in M2 macrophages through a lysosomal calcium channel transient receptor potential cation channel mucolipin 1 (TRPML1) (1). This report suggested that lysosomal inhibition induces TRPML1-dependent calcium release, which leads to phosphorylation of p38 (p-p38), and p–NF-κB p65 that in turn regulates the transcriptional profile of macrophages that manifests in the M2 to M1 phenotype switch. In M2 polarized macrophages, HCQ or DC661 produced significant intracellular calcium release (Figure 5A). HCQ, DC661, or the weak PPT1 inhibitor hexadecylsulfonyl fluoride (HDSF) treatment resulted in increased levels of p-p38 (Figure 5B). In addition, KD of *Ppt1* also resulted in increased levels of p-p38 compared with siNon-target control (Figure 5C). PPT1 inhibition with HCQ or DC661 induced phosphorylation of p–NF-κB p65, a gatekeeper transcription factor for the proinflammatory phenotype (Figure 5D). Inhibition of p38 completely abrogated the M2 to M1 phenotype switch elicited by treatment with HCQ or DC661 (Figure 5E).

To understand how PPT1 inhibition was inducing phosphorylation of p38, we first determined if calcium release following Ppt1 inhibition was required for phosphorylation of p38. Cotreatment of M2 polarized macrophages with Ppt1 inhibitors and BAPTA-AM, a calcium chelator (Figure 5F), or W7, a calmodulin inhibitor (Figure 5G), abrogated HCQ- or DC661-induced phosphorylation of p38. However, contradicting a previous report (1), we found that verapamil, a TRPML1 calcium channel blocker, was not able to abrogate HCQ- or DC661-induced phosphorylation of p38 (Figure 5H). As a positive control, the TRPML1 agonist MK6-83 also induced phosphorylation of p38, which was completely abrogated by TRPML1 inhibitor verapamil. Upstream of TRPML1 is the lipid kinase PIKfyve, which phosphorylates phosphatidylinositol [PtdIns(3)P] to PtdIns(3,5)P2. TRPML1 activity requires binding to PtdIns(3,5)P2, the lipid product of PIKfyve (16). PIKfyve inhibition with vacuolin-1 was not able to blunt HCQ- or DC661-induced or MK6-83– induced phosphorylation of p38 (Figure 5I). Since these experiments determined that TRPML1 was not likely responsible for the calcium release in macrophages treated with Ppt1 inhibition, we next investigated other subcellular compartments that could be responsible for the calcium accumulation found with Ppt1 inhibition. The ER calcium channel blocker ryanodine was unable to prevent DC661-induced activation of p-p38. However, CGP37157, an inhibitor of the mitochondrial Na^+^/Ca^2+^ exchanger, completely abrogated the increased p38 phosphorylation induced by lysosomal inhibitors (Figure 5J). These results support that lysosomal inhibition is associated with calcium release from the mitochondria and activation of p38, which is required for macrophage phenotype switching.

**Figure 6:**
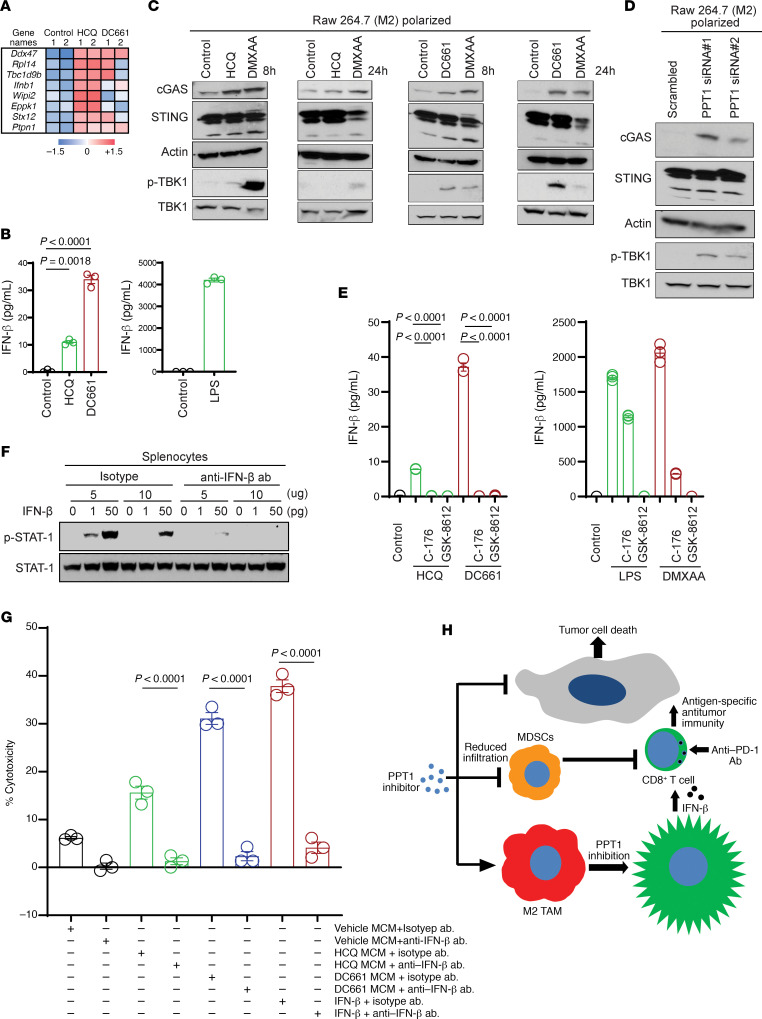
PPT1 inhibition stimulates IFN-β secretion by macrophages via activation of cGAS/STING/TBK1 pathway and enhances T cell antitumor activity. (**A**) Relative mass spectrometry signal of proteins in control, HCQ-treated, or DC661-treated MCM that showed a 5-fold increase and *P* < 0.05 in HCQ-treated MCM relative to control. (**B**) Dot plot representing ELISA performed for the measurement of IFN-β in HCQ- or DC661-treated MCM. Each result was obtained in duplicate, and the results were reproduced with 3 independent experiments. (**C**) Immunoblots showing cGAS, STING, and p-TBK1 protein status in macrophages treated with HCQ, DC661, or DMXAA for the time indicated. (**D**) Immunoblots for cGAS, STING, and p-TBK1 proteins in *Ppt1*-KD macrophages. (**E**) Dot plots representing ELISA performed for the measurement of IFN-β in HCQ- or DC661-treated or STING inhibitor C-176 or TBK1 inhibitor GSK-8612 cotreated MCM. Each result was obtained in duplicates, and the results were reproduced with 3 independent experiments. (**F**) Immunoblot showing the status of p-STAT1 in isotype or anti–IFN-β neutralizing Ab in the presence or absence of recombinant IFN-β in mouse splenocytes. (**G**) Percentage cytotoxicity elicited by primed splenocytes with exposure to MCM collected from RAW 264.7 macrophages treated with control, HCQ, or DC661 along with the administration of isotype or anti–IFN-β neutralizing Ab as indicated. Each result was obtained in duplicates, and the results were reproduced with 3 independent experiments. (**H**) Schematic showing the PPT1 inhibition results in reduced MDSCs tumor infiltrations and macrophage M2 to M1 switching. Polarized macrophages upon PPT1 inhibition secrete IFN-β, which enhances the CD8^+^ T cell–mediated tumor cell killing. One-way ANOVA and Dunnett’s procedure (**B** and **E**) or Tukey’s procedure (**G**).

For an unbiased approach to identify how macrophage activation following Ppt1 inhibition could lead to augmented T cell killing, we performed secretome analysis on M2 polarized macrophages grown in 0.01% serum. First, we confirmed under these conditions that conditioned medium from macrophages treated with HCQ or DC661 was still able to augment antigen-primed T cell killing (data not shown). MCM was subjected to proteome analysis. Comparison of HCQ versus control showed 64 significantly changed proteins (fold change > 2 and *P* value < 0.05), while a comparison of DC661 versus control showed 47 significantly changed proteins out of a total of 3166 proteins identified with high confidence (Figure 6A).

We focused our attention on IFN-β, since it has been previously linked to T cell function (17). PPT1 inhibition by HCQ- or DC661-treated M2 macrophages produced a significant increase in secreted IFN-β secretion compared with vehicle control (Figure 6B). IFN-β production can be increased with the activation of the cGAS/STING/TBK1 pathway (18). We observed that the Ppt1 inhibitors HCQ and DC661 induced increased expression of cGAS in 8 and 24 hours. Unlike DMXAA, a known STING ligand that leads to degradation of STING at 24 hours, HCQ or DC661 treatment instead resulted in sustained expression of STING protein. Phosphorylation of TBK1 was detected transiently at 8 hours following HCQ treatment and more persistently at 8 and 24 hours following DC661 treatment (Figure 6C). *Ppt1* KD mimicked HCQ and DC661 treatment and showed similar increase in cGAS and p-TBK1 induction and maintained STING level (Figure 6D). The role of PPT1 inhibition by HCQ and DC661 induced cGAS/STING/TBK1 upregulation, and subsequent IFN-β production was further supported when cotreatment with STING inhibitor C-176 or TBK1 inhibitor GSK-8612 resulted in abrogation of IFN-β production by HCQ and DC661 or DMXAA (Figure 6E). Next, we optimized an anti–IFN-β neutralization antibody. We treated mouse splenocytes with exogenous IFN-β in the absence and presence of neutralizing antibody and measured p-STAT1 as a readout of IFN-β activity (Figure 6F). Finally, we repeated the primed T cell–tumor cell coculture experiment as described in Figure 3E. Antigen-primed T cells exposed to conditioned medium from M2 polarized macrophages treated with HCQ, or DC661, exhibited significantly better tumor cell killing than control. This augmentation of cell killing was completely abrogated when anti–IFN-β antibody was coadministered with the conditioned medium (Figure 6G).

## Discussion

Our findings show that PPT1 inhibition likely augments tumor immunity by at least 3 means: by causing an M2 to M1 macrophage polarization switch, by reducing the number of MDSCs in the tumor microenvironment, and by inducing IFN-β release from macrophages that stimulates T cell–mediated killing (Figure 6H). We also found a significant increase in the infiltration of NK cells into the tumor microenvironment (Supplemental Figure 4, B and C), which could also be directly related to IFN-β production induced by CQ derivatives. There is a large body of literature that demonstrates that macrophages produce type I IFNs, such as IFN-β (17), and that type I IFNs directly activate CD8^+^ cytolytic T cells (19–21). These studies and the results of our studies demonstrate that macrophages secrete 1–30 pg/mL of IFN-β, upon stimulation, but this is enough to augment cytotoxic activity of antigen-primed T cells. In the combination of anti–PD-1 Ab and PPT1 inhibitor, the anti–PD-1 Ab is likely reinvigorating the T cells and the PPT1 inhibition is further augmenting the efficacy of these antigen-primed T cells through macrophage-dependent type I IFNs.

Unlike Chen et al. we did not see single-agent antitumor activity with HCQ in 2 mouse models of melanoma. This is in line with the lack of evidence of single activity of HCQ in clinical melanoma. Although numerous reports have shown that lysosomal inhibition or deletion of autophagy genes can impair tumor growth in MAPK-mutant cancers (22) as single agents, in some contexts, deletion of autophagy genes, such as *Atg7*, is well tolerated by tumor cells, and in most models HCQ does not produce single-agent antitumor activity (23). Previous findings suggest that *Atg7* depletion leads to enhanced MHC class I presentation in tumor cells but reduced MHC class II expression (23). A recent paper showed that in pancreatic cancer autophagy inhibition can upregulate MHC class I and recruit T cells into the tumor microenvironment. In this pancreatic cancer study, autophagy inhibition with CQ or genetic autophagy inhibition was unable to augment anti–PD-1 Ab antitumor activity but significantly augmented the activity of combined anti–PD-1 Ab and anti–cytotoxic T lymphocyte–associated protein 4 antibody (24). In our melanoma study we see clear augmentation of T cell priming with PPt1 inhibition but not with genetic inhibition of upstream autophagy genes. B16 expresses low levels of MHC class I and MHC class II, and the studies here suggest that priming of CD8^+^ T cells by irradiated B16 may require MHC class II expression (25). It is important to note that although the in vivo findings in B16 tumors are only with *Atg7*-KO conditions, given the lack of efficacy in T cell priming and antigen-primed T cell–mediated cytotoxicity when any of the core autophagy genes *Ulk1*, *Vps34*, or *Atg7* were knocked down in vitro, it is also unlikely that PD-1 treatment would be more effective in tumors deficient in these autophagy genes. Since Ulk1, Vps34, and Atg7 represent the key nodes of the autophagy pathway, these results indicate that the effects of Ppt1 inhibitors are independent of the autophagy pathway in tumor cells at least in the B16 model.

Here we chose B16 and the *BRaf^CA^ Pten^loxP^ Tyr::CreER^T2^* genetically engineered mouse models as examples of immune “cold” tumors that are poorly responsive to anti–PD-1 Ab. This is a group of tumors sorely in need of new therapies and therefore relevant models for our purpose. While the combination of anti–PD-1 and PPT1 inhibition by HCQ did not result in significant change in the number of activated T cells as assessed by immunophenotyping, the clear changes in macrophage populations could also have a significant impact on antitumor activity. Our finding that PPT1 inhibition leads to calcium release warrants further study, which we have started in our laboratory. It is known that TRPML1 is a palmitoylated protein, and in humans mutation or loss of this protein leads to a lysosomal storage disorder with a similar phenotype as mutations or loss of *Ppt1*. Therefore, it may be that PPT1 directly regulates TRPML1. Our study shows that TRPML1-independent calcium release and activation of p-p38 is required for PPT1 inhibitor–mediated secretion of IFN-β by macrophages. This calcium release may be critical also for the susceptibility of MDSCs to lysosomal inhibition. MDSCs express high levels of calcium-dependent proteins, such as S100A8/A9. Further work is needed to understand how PPT1 inhibitor–induced calcium release regulates MDSC viability. It has been previously reported that activation of the cGAS/STING/TBK1 pathway leads to the degradation of the STING protein via the lysosome (26). Therefore, PPT1 inhibition with HCQ and DC661 in our study with no observed degradation of STING protein strongly supports that sustained cGAS/STING/TBK1 signaling and IFN-β production is the mechanism by which these agents enhanced both CD8^+^ T cell activity and the efficacy of anti–PD-1 in these models. Given the excellent tolerability of HCQ combined with either targeted (5) or chemotherapy regimens (6), there is now sufficient rationale to launch clinical trials of combined CQ derivatives and anti–PD-1 Ab. Our group has launched the Lysosomal Inhibition + Melanoma ImmunoTherapy melanoma trial (ClinicalTrials.gov NCT04464759).

## Methods

### Reagents.

DC661 was provided in-house. Purity of the sample was determined by NMR spectroscopy and liquid chromatography-mass spectrometry. Mouse melanoma B16-F10 (CRL-6475) and RAW 264.7 (TIB-71) were purchased from ATCC. Cell lines were tested for mycoplasma biannually and authenticated using short-tandem repeat fingerprinting. *Atg7*-KO B16 cells were generated using CRISPR/Cas9 editing. *PpT1* siRNAs pool 1 (MilliporeSigma EMU085041) CAGCATCTTCTTGGCAGACATAAATCAAGAGAGGTGTGTCAATGAGTCCTACAAGAAGAACCTGATGGCCCTCAAGAAGTTTGTGATGGTGAAATTCTTTAATGATTCCATTGTGGACCCTGTCGACTCTGAGTGGTTTGGATTTTACAGAAGTGGCCAAGCTAAGGAAACCATTCCCCTCCAGGAGAGCACTCTATACACAGAGGACCGCCTGGGGCTAAAGAAAATGGACAAAGCAGGAAAGCTAGTGTTTCTGGCTAAGGAAGGGGACCATCTTCAAATATCTAAAGAATGGTTTACTGCCCACATCATACCTTTTCTTAAGTGATGCCCTGGCACTTTATAGCAGAGTTCATGAAACCACAGCTCTTCCAAGCCATGTACATAGTTCATGCTCAGCCTGAACTCTAATCTAGCCTGCAACCAGCCCTTCTCTCCTCTTATCATCTAACATACCCTACTTGGAAAGATCTAAGATCTCAATCTTATCCTTTGCCGCCT. *PpT1* siRNA pool 2 (Santa Cruz Biotechnology sc-142398) AGGCGGCAAAGGATAAGATTGAGATCTTAGATCTTTCCAAGTAGGGTATGTTAGATGATAAGAGGAGAGAAGGGCTGGTTGCAGGCTAGATTAGAGTTCAGGCTGAGCATGAACTATGTACATGGCTTGGAAGAGCTGTGGTTTCATGAACTCTGCTATAAAGTGCCAGGGCATCACTTAAGAAAAGGTATGATGTGGGCAGTAAACCATTCTTTAGATATTTGAAGATGGTCCCCTTCCTTAGCCAGAAACACTAGCTTTCCTGCTTTGTCCATTTTCTTTAGCCCCAGGCGGTCCTCTGTGTATAGAGTGCTCTCCTGGAGGGGAATGGTTTCCTTAGCTTGGCCACTTCTGTAAAATCCAAACCACTCAGAGTCGACAGGGTCCACAATGGAATCATTAAAGAATTTCACCATCACAAACTTCTTGAGGGCCATCAGGTTCTTCTTGTAGGACTCATTGACACACCTCTCTTGATTTATGTCTGCCAAGAAGATGCTG. *Ppt1* siRNA single duplex (OriGene, SR409088) CTGGTTGCAGGCTAGATTAGAGTTCAGGCTGAGCATGAACTATGTACATGGCTTGGAAGAGCTGTGGTTTCATGAACTCTGCTATAAAGTGCCAGGGCATCACTTAAGAAAAGGTATGATGTGGGCAGTAAACCATTCTTTAGATATTTGAAGATGGTCCCCTTCCTTAGCCAGAAACACTAGCTTTCCTGCTTTGTCCATTTTCTTTAGCCCCAGGCGGTCCTCTGTGTATAGAGTGCTCTCCTGGAGGGGAATGGTTTCCTTAGCTTGGCCACTTCTGTAAAATCCAAACCACTCAGAGTCGACAGGGTCCACAATGGAATCATTAAAGAATTTCACCATCACAAACTT. Fluo-4, AM, was used to stain the cells for calcium as per the manufacturer’s instructions (Thermo Fisher Scientific, F14201).

### Cell culture.

Cells were cultured in Dulbecco’s modified Eagle medium (DMEM) supplemented with 4.5 g/L glucose, sodium pyruvate, phenol red, and 10% fetal calf serum (MilliporeSigma, F6178). BMDMs were isolated as described previously (1) and cultured as above in DMEM in the presence of mouse macrophage CSF, 10 ng/mL. RAW 264.7 macrophages or BMDMs were M1 polarized with mouse IFN-γ (10 ng/mL) and LPS (100 ng/mL) or M2 polarized with mouse IL-4 (10 ng/mL) and IL-13 (10 ng/mL) for 24 hours. Splenocytes were isolated as described previously (2).

### qPCR and primers.

Total RNA was isolated by RNA isolation kit (QIAGEN, 74134) according to the manufacturer’s protocol. Complementary DNA (cDNA) was synthesized using iScript Reverse Transcriptase kit with 500 ng of purified RNA as per manufacturer’s protocol (Bio-Rad, 1708890). The qPCR reaction was set up using SYBR Green PCR Master Mix (Bio-Rad, 1725121) containing 1 μL of cDNA. All measurements were carried out in duplicate, and Hsp90 was used as internal standard for ΔCT calculations. Gene expression analysis was done using the following primers: iNos forward (AGGAGGAGAGAGATCCGATTTAG), iNos reverse (TCAGACTTCCCTGTCTCAGTAG); Retnla/Fizz1 forward (TGCCAATCCAGCTAACTATCC), Retnla/Fizz1 reverse (GCAAAGCCACAAGCACAC); Hsp90 forward (GGGAGCTCATCTCCAATTCATC), Hsp90 reverse (GTCCTGTTTGCTGGGAATGA); Arg1 forward (TACCTGCTGGGAAGGAAGAA), and Arg1 reverse (CTGTAAGATAGGCCTCCCAGA).

### CRISPR/Cas9 editing.

The nontargeting guide RNA (gRNA) TAGCGAACGTGTCCGGCGT and *Atg7* gRNA AACTCCAACGTCAAGCGGGT sequences for targeting mouse cells were used as described previously (3). The 2 separate plasmid constructs pCW-Cas9 (Addgene ID 50661) for the expression of the inducible-hSpCas9 and pLX-sgRNA (Addgene ID 50662) for the expression of targeting gRNA were used based on the protocol described previously (4). Subcloning of the target gRNA sequence into the pLX-sgRNA vector was performed by 3 PCR extension steps within the *Xho*I and *Nhe*I sites of pLX-sgRNA followed by restriction digest and ligation. Sanger sequencing was used to confirm that the *Atg7* gRNAs and nontargeting control RNA were correctly subcloned in the pLX-sgRNA vector. B16 cells were first transfected with the pCW-Cas9 vector and selected with puromycin 4 μg/mL for 6 days and then transfected with the pLX-sgRNA containing the *Atg7* gRNA and nontargeting gRNA using Lipofectamine 3000 (Thermo Fisher Scientific) based on the manufacturer’s instructions. After 48 hours’ incubation, growth medium was changed to selection medium containing blasticidin (Thermo Fisher Scientific, catalog A1113902) 6 μg/mL. After blasticidin selection for 12 days, the cells were treated with doxycycline 0.5–1 μg/mL to express Cas9 and induce *Atg7* deletion. Doxycycline was replenished every 2 days for 2 weeks, after which the cells were harvested and analyzed by immunoblotting for *Atg7* deletion (Figure 1A).

### Immunoblotting.

Whole-cell lysates and lysosomal extracts were immunoblotted as previously described (5, 6).

### In vivo studies.

Tumor generation, measurement, and harvesting were performed as previously described (7, 9). Briefly, B16 (mouse melanoma) cells (ATCC, catalog CRL-6475) were subcutaneously injected (5 × 10^5^) with an equal volume of Matrigel (Corning, 354234) in the right flank of C57BL6/J mice. Daily injection of HCQ (60 mg/kg) and every-other-day injection of anti–PD-1 Ab (200 μg) commenced at the tumor size of 50 mm^3^. *BRaf^CA^ Pten^loxP^ Tyr::CreER^T2^* mice (The Jackson Laboratory) were treated topically on the back with 4-HT to elicit *BRaf^V600E^* and to silence *Pten* expression. Tumors were measured using electric calipers (Digital Caliper, model H-7352). Tumor volume was calculated as L × W^2^ × 0.5. In all animal experiments 2-tailed *t* test or 2-tailed *t* test for unequal variance was used to test the hypothesis that the addition of HCQ to anti–PD-1 Ab is significantly different compared with anti–PD-1 Ab + Veh.

### Tumor digestion.

Tumor was excised and digested with a tumor digestion kit (Miltenyi Biotec, catalog 130-096-730) according to the manufacturer’s protocol.

### Flow cytometry and IHC.

Cells (1 × 10^6^) from the digested tumor were stained with antibodies (please see Supplemental Table 1). Stained samples were acquired on a BD LSRII flow cytometer, and the gating strategy was followed as in Supplemental Figure 4A and Supplemental Figure 5A. Mouse tumor IHC was performed as per standard protocol.

### T cell priming and percentage cytotoxicity.

B16 tumor cells (5 × 10^5^) were cultured and irradiated with 25 Gy x-rays. Splenocytes were then cultured with (primed) or without (unprimed) irradiated B16 cells in the presence of IL-2 (5 IU/mL) and cocultured for 48 hours. Splenocytes cultured with concanavalin A (10 μg/mL) were used as a nonspecific T cell priming control. Priming was confirmed by IFN-γ ELISA of the supernatant. Primed splenocytes were then cocultured with freshly cultured B16 cells with various target (B16) to effector (splenocytes) ratios. The B16 cell death–associated LDH release and then percentage cytotoxicity were measured according to the manufacturer’s protocol (MilliporeSigma, catalog 4744926001).

### Macrophage-conditioned medium.

MCM was generated by treating M2 polarized RAW 264.7 macrophages with vehicle or DC661 for 6 and 24 hours. Supernatant was spun at 672*g* for 3 minutes to remove any viable or dead macrophages, and then MCM was added to fresh B16 cells cocultured with primed or unprimed splenocytes. LDH release and percentage cytotoxicity were measured.

### Proteomics and secretome analysis.

MCM from M2 polarized macrophages grown in 0.01% serum was centrifuged (672*g*) to remove cellular debris. The supernatant was passed through a 0.2 μm filter, concentrated 50-fold using an Amicon Ultra 3K membrane, electrophoresed for 0.5 cm into an SDS-PAGE gel, and stained with colloidal Coomassie (Invitrogen, Thermo Fisher Scientific, catalog LC6025). The entire stained region was excised, digested with trypsin, and analyzed by liquid chromatography-tandem mass spectrometry on a Q Exactive HF mass spectrometer (Thermo Fisher Scientific) coupled with a Nano-ACQUITY UPLC system using a 245-minute gradient and acquisition parameters as previously described (24). RAW files were searched using MaxQuant 1.5.3.30 using default parameters, and peptide sequences were identified against a mouse UniProt database (October 2019) using full tryptic specificity, up to 2 missed cleavages, fixed modification of carbamidomethyl (Cys), and variable Met oxidation and protein N-terminal acetylation. An in-house common contaminant database composed of trypsin, keratins, bovine proteins found in serum, and mycoplasma proteins was appended to the mouse sequence database. Protein and peptide false discovery rates were set at 1%. Proteins identified by at least 2 peptides were analyzed using label-free quantitation to determine the ratio of protein intensity in HCQ- or DC661-treated MCM versus control.

### Statistics.

For continuous variables, a 2-sample *t* test was used to test the primary hypothesis that there was a significant difference between the 2 experimental groups receiving either HCQ and anti–PD-1 Ab or anti–PD-1 Ab + Veh. For experiments comparing more than 2 groups, continuous variables were analyzed using 1-way ANOVA with adjusted *P* values using Dunnett’s procedure when each experimental group was compared with control or Tukey’s procedure when all pairwise comparisons were considered. For relative expression, a 1-sample *t* test was used to test the hypothesis that mean differences were different from 1 with adjusted *P* values computed using the Holm-Bonferroni procedure. For survival times, an exact log-rank test was used to compare the survival curves between the HCQ and anti–PD-1 Ab or anti–PD-1 Ab + Veh groups. Growth rates (mm^3^/d) were computed for each mouse using linear regression of the natural logarithm of tumor volume on time; the estimated slopes for the HCQ and anti–PD-1 Ab or anti–PD-1 Ab + Veh groups were analyzed using a 2-sample *t* test. All *t* tests were 2 tailed. A *P* value less than 0.05 was considered significant. *P* values presented in the figures are for the test of the hypothesis that the expected mean when HCQ is added to anti–PD-1 Ab is significantly different from the expected mean for anti–PD-1 Ab + Veh. Adjusted *P* values are indicated as * when the adjusted *P* < 0.05 and as ^ when the adjusted *P* < 0.10. All analyses were done using SAS/STAT software, version 9.4 of the SAS system for Windows.

### Study approval.

All animal experiments were performed in accordance with the protocols approved by the University of Pennsylvania Institutional Animal Care and Use Committee.

## Author contributions

GS, RO, ENO, EJW, DG, XX, DWS, PAG, and RKA conceived experiments. GS, RO, ENO, JM, JA, VWR, SL, SP, JLL, SH, AR, VJ, MCN, JDW designed the experiments, conducted the experiments, and collected the data. GS, XX, ENO, EJW, DG, JDW, DWS, PAG, and RKA supervised experiments and analyzed the data. GS, DWS, DG, PAG, and RKA wrote the manuscript with assistance from all the other authors.

## Supplementary Material

Supplemental data

## Version 1 08/11/2020

In-Press Preview

## Version 2 09/03/2020

Electronic publication
