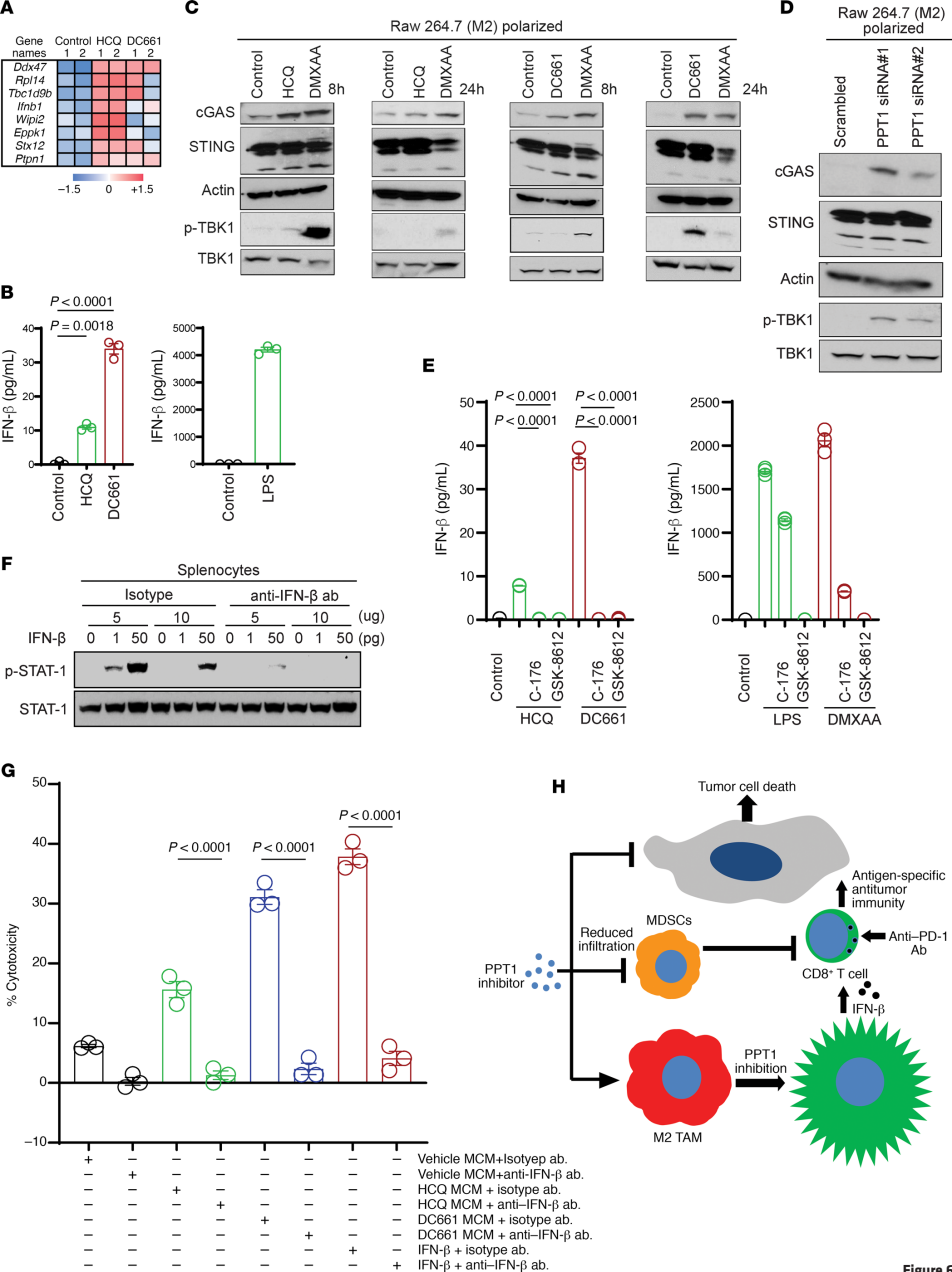# PPT1 inhibition enhances the antitumor activity of anti–PD-1 antibody in melanoma

**DOI:** 10.1172/jci.insight.165688

**Published:** 2022-10-24

**Authors:** Gaurav Sharma, Rani Ojha, Estela Noguera-Ortega, Vito W. Rebecca, John Attanasio, Shujing Liu, Shengfu Piao, Jennifer J. Lee, Michael C. Nicastri, Sandra L. Harper, Amruta Ronghe, Vaibhav Jain, Jeffrey D. Winkler, David W. Speicher, Jerome Mastio, Phyllis A. Gimotty, Xiaowei Xu, E. John Wherry, Dmitry I. Gabrilovich, Ravi K. Amaravadi

Original citation: *JCI Insight*. 2020;5(17):e133225. https://doi.org/10.1172/jci.insight.133225

Citation for this corrigendum: *JCI Insight*. 2022;7(20):e165688. https://doi.org/10.1172/jci.insight.165688

The authors recently became aware that one of the p-TBK1 blot images presented in Figure 6C is the same as that presented in Figure 6D. The authors reviewed the original data and determined that the image in Figure 6C was incorrect. The correct version of Figure 6C is shown below, and the HTML and PDF versions have been updated.

The authors regret the error.

## Figures and Tables

**Figure 6:**