# Supplementary material for: PPT1 inhibition enhances the antitumor activity of anti–PD-1 antibody in melanoma
Source: JCI Insight. 2020 Sep 3;5(17):e133225. doi: 10.1172/jci.insight.133225 (PMC7526447; doi:10.1172/jci.insight.133225)
Supplement: Supplemental data [file jciinsight-5-133225-s091.pdf]

Supplementary Figures

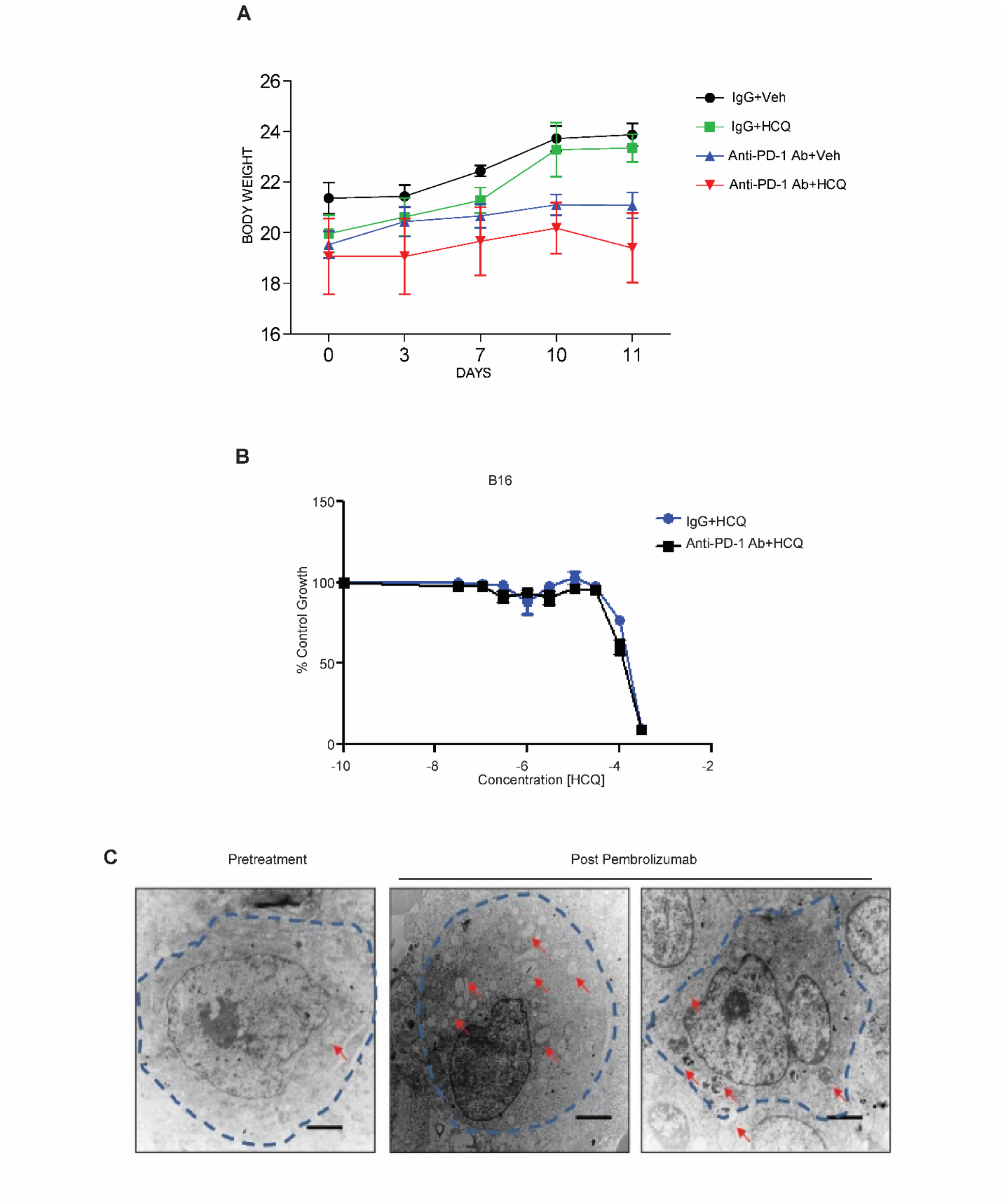

Supplementary Fig. S1. **B16 tumor model and Melanoma patient biopsies. (A)** Measurement of mice body weight during drug treatment. **(B)** Fraction survival of B16 tumor bearing mice during the treatment. **(C)** Electron microscopy of tumor biopsies before and on pembrolizumab treatment in a stage IV melanoma patient that progressed on pembrolizumab. *, p< 0.05, testing the hypothesis that the addition of HCQ to anti-PD-1 Ab is significantly different compared to anti-PD-1 Ab+Veh.

Supplementary Figures

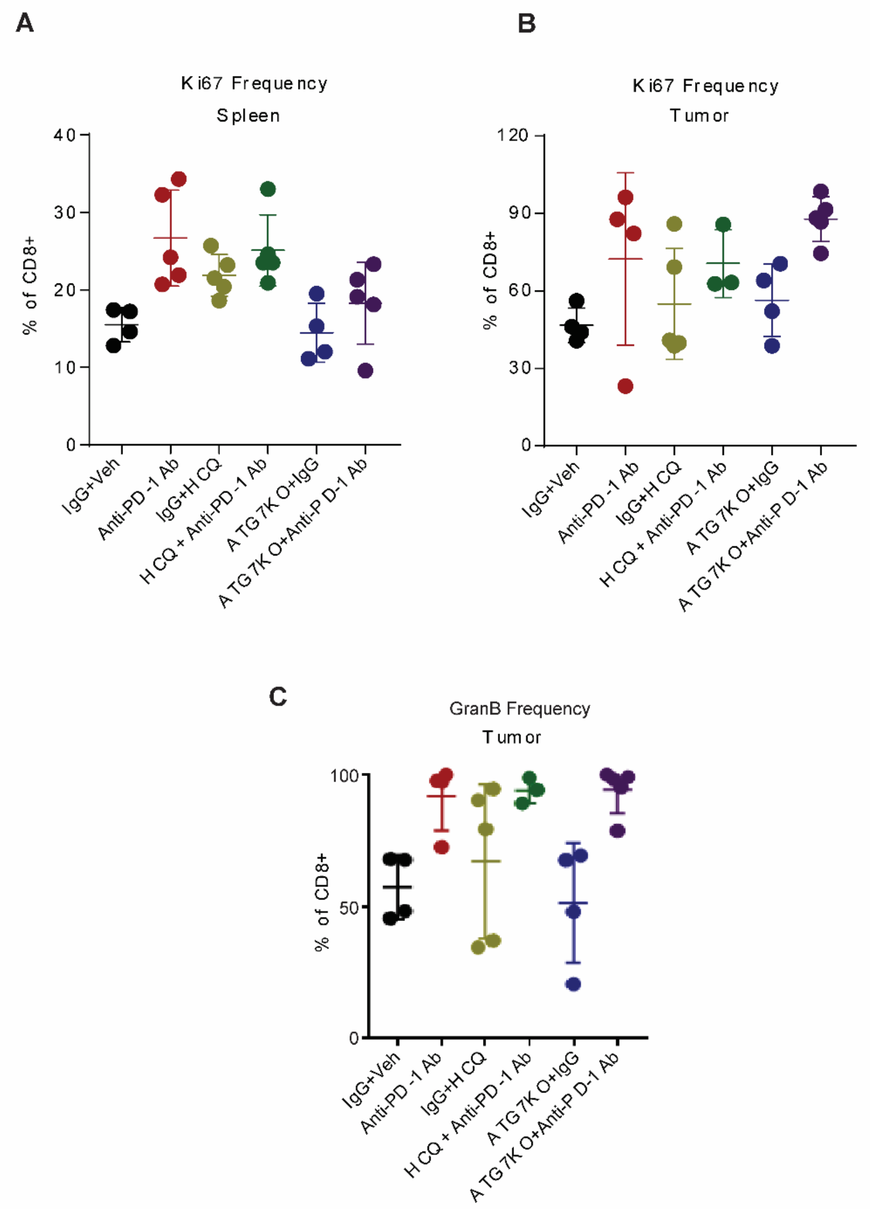

Supplementary Fig. S2. Immunophenotyping of CD8+ T cells in the spleen and tumor of B16 *Atg7* WT and *Atg7* KO mice and LDH release in inhibitor treated or untreated primed splenocytes. (A) Frequency of CD8+Ki67+ T cells in the spleen of tumor bearing mice of the indictaed treatment groups. **(B)** Representation of the frequency of CD8+Ki67+ T cells in the spleen of tumor bearing mice of the indictaed treatment groups. **(C)** Frequency of CD8+GranzymeB+ T cells in tumor. *, $p < 0.05$, testing the hypothesis that the addition of HCQ to anti-PD-1 Ab is significantly different compared to anti-PD-1 Ab+Veh.

Supplementary Figures

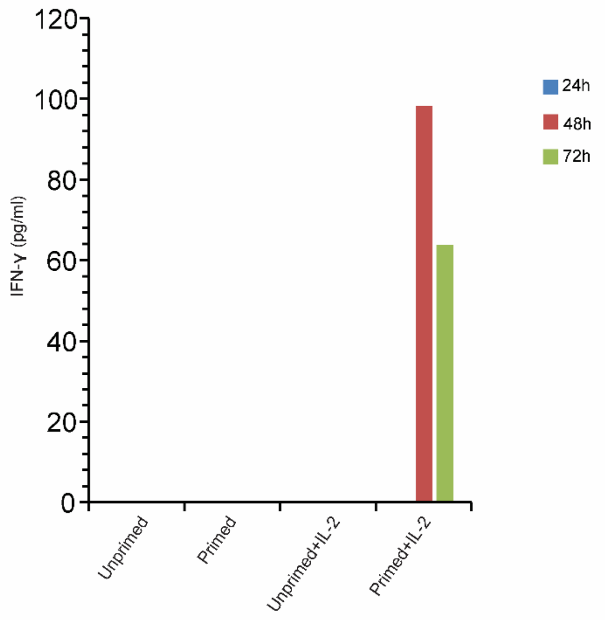

Supplementary Fig. S3. Splenocyte priming. IFN-γ ELISA to confirm irradiated B16 mediated splecnocytes priming in the presence or absence of interleukin-2 for 24, 48 and 72h

Supplementary Figures

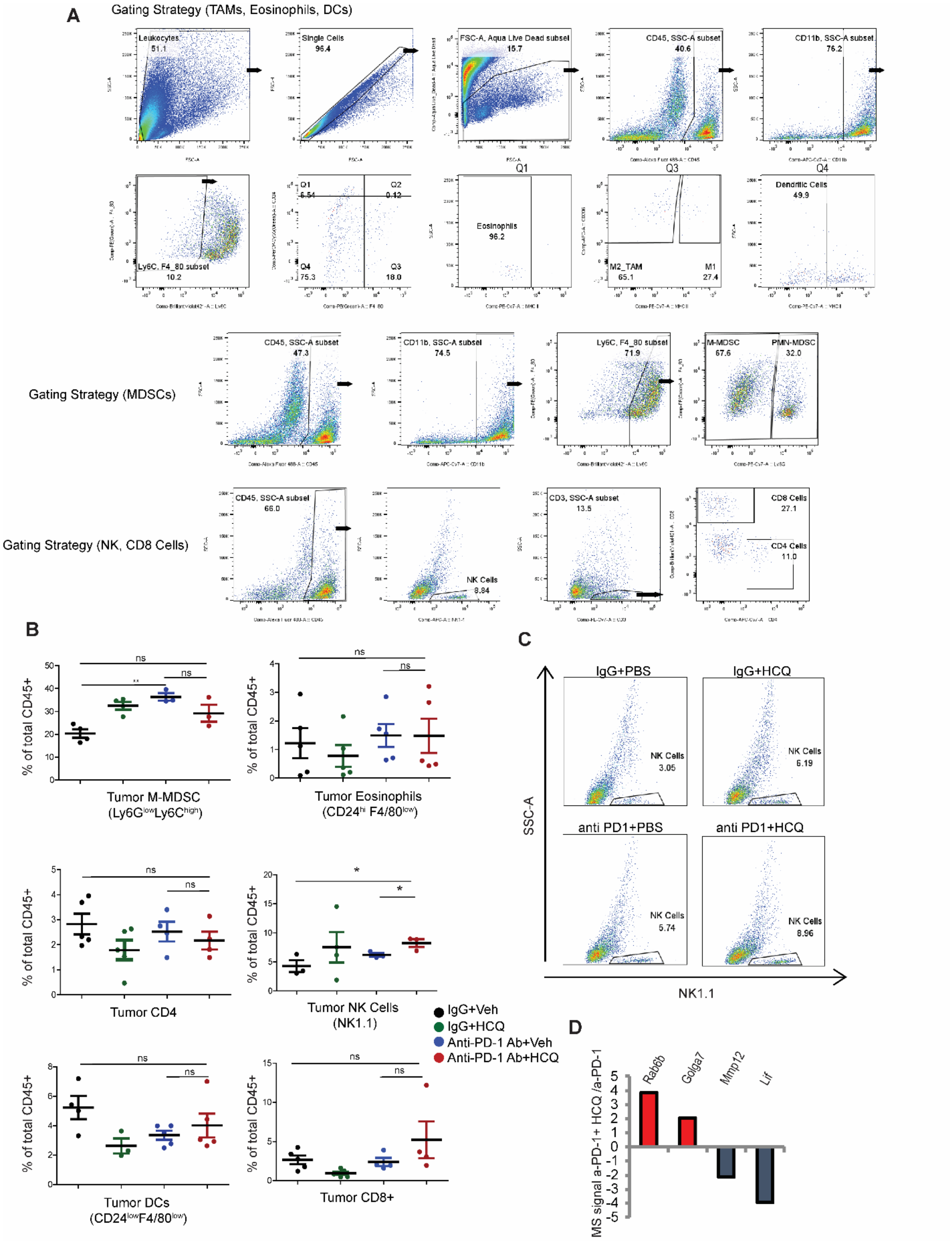

Supplementary Fig. S4. **Gating Strategy for Flowcytometry and immunophenotyping of B16 tumors and proteomics of whole B16 tumor**. **(A)** flowcytometry analysis and gating strategy for TAMs, Eosinophils, DCs, MDSCs, CD8, NK cells in B16 tumor. **(B)** Representation immunophenotyping plot for Tumor M-MDSCs, eosinophils, DCs, CD8, CD4 and NK cells. **(C)** Representation of Immunophenotyping of TILs in B16 Tumors. **(D)** Identification of macrophages proteins that are significantly increased or decreased in HCQ+anti-PD-1 Ab vs only anti-PD-1 Ab+Veh. *, p< 0.05, testing the hypothesis that the addition of HCQ to anti-PD-1 Ab is significantly different compared to anti-PD-1 Ab+Veh.

Supplementary Figures

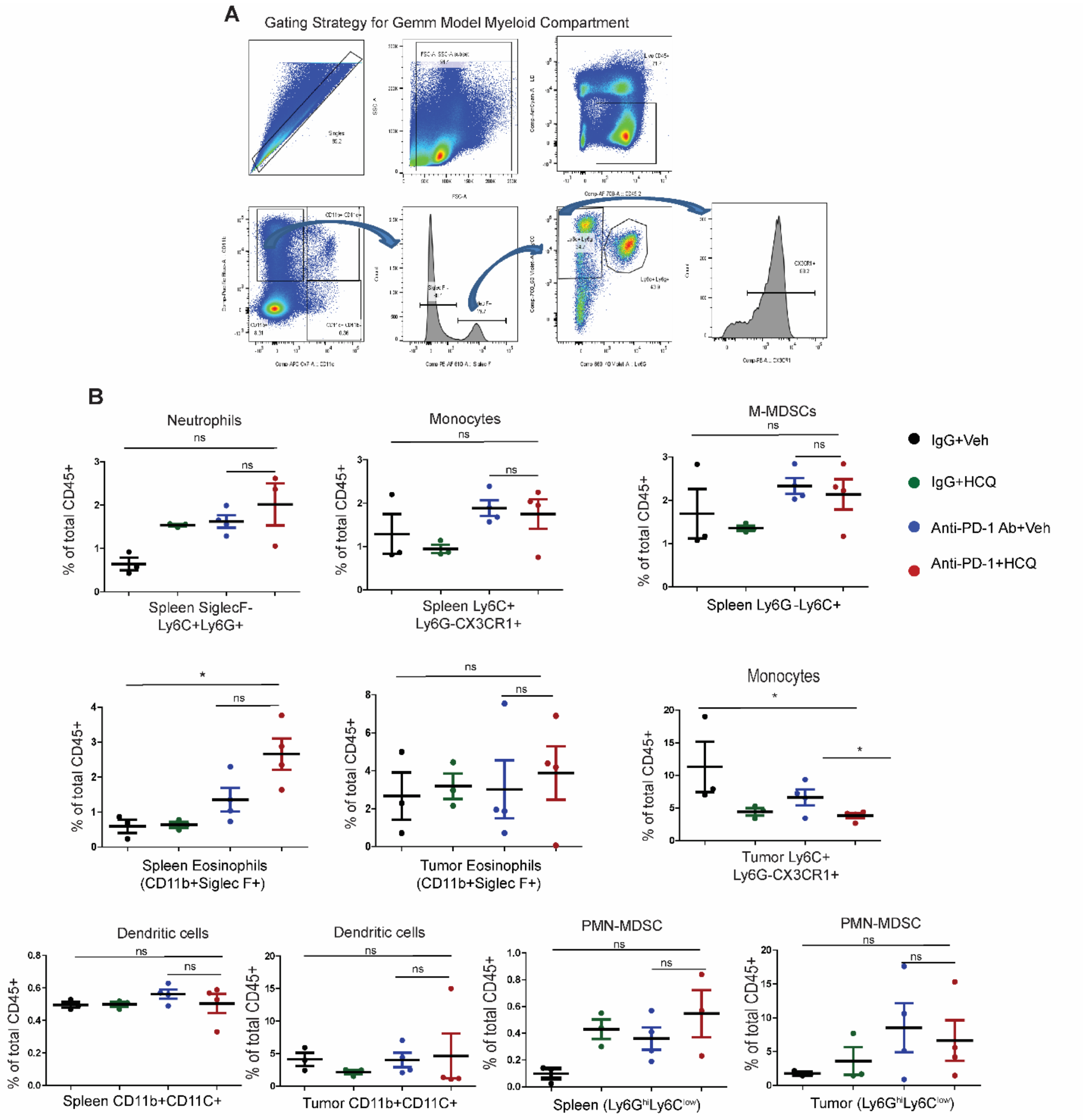

Supplementary Fig. S5. Gating strategy for Flowcytometry and Immunophenotying in Braf ^{CA}, Pten$^{flox/flox}$, Tyr::CreER^{T2} mice melanoma tumor.
(A) Gating strategy for immunophenotyping in *Braf* ^{CA}, *Pten$^{flox/flox}$*, *Tyr::CreER^{T2}* mice melanoma. **(B)** Representation of Immunophenotyping of tumor infiltrating myeloid cells in the excised tumor as indicated. *, p< 0.05, testing the hypothesis that the addition of HCQ to anti-PD-1 Ab is significantly different compared to anti-PD-1 Ab+Veh.

Supplementary Figures

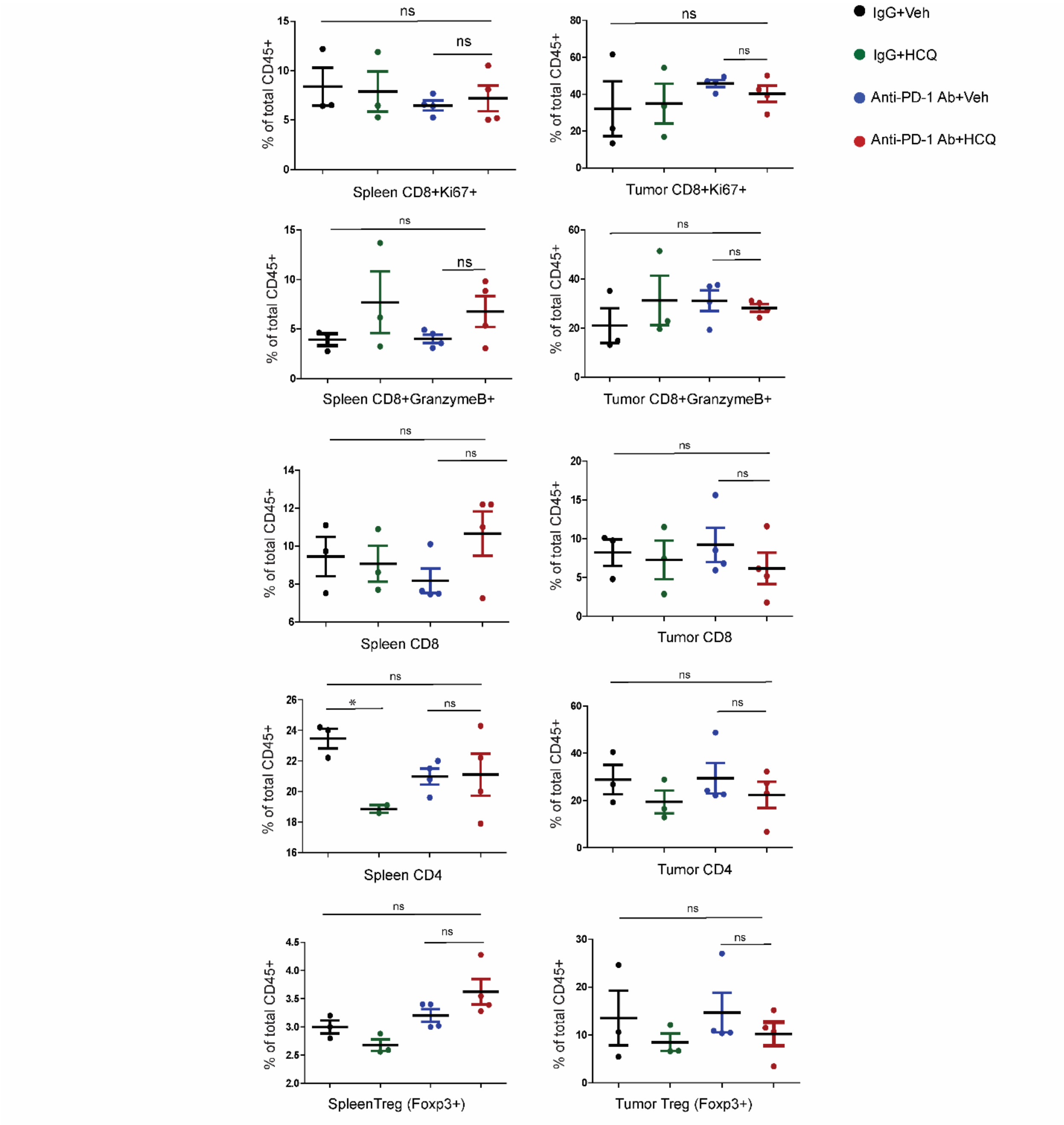

Supplementary Fig. S6. Immunophenotyping in Braf ^{CA}, Pten$^{flox/flox}$, Tyr::CreER^{T2} mice melanoma. Immunophenotyping of tumor infiltrating leukocytes in excised tumor of *Braf ^{CA}, Pten$^{flox/flox}$, Tyr::CreER^{T2}* mice. *, $p < 0.05$, testing the hypothesis that the addition of HCQ to anti-PD-1 Ab is significantly different compared to anti-PD-1 Ab+Veh.

Table 1

S. No.	Source	Reagent or Resource	Identifier
1	BD Biosciences	CD11b-APC-Cy7	557657
2	BD Biosciences	Ly-6C-BV421	562727
3	BD Biosciences	Ly-6G-PE-Cy7	560601
4	BD Biosciences	CD45-FITC	553080
5	BD Biosciences	CD11c-APC	550261
6	BD Biosciences	NK1.1-APC	550627
7	BD Biosciences	Foxp3-PE	560408
8	BD Biosciences	CD3-PE-Cy7	552774
9	BD Biosciences	CD24-PerCP-Cy5.5	562360
10	BD Biosciences	CD16/CD32 purified	553142
11	BD Biosciences	Mouse Foxp3 Buffer Set	560409
12	BD Biosciences	CD3e-BV605	563004
13	BD Biosciences	Ly6G-BV650	740554
14	BD Biosciences	CTLA-4-PE-CF594	564332
15	BioLegend	Anti-PD-1 Ab	92130
16	BioLegend	CD8-BV421	100753
17	BioLegend	CD11b- BV421	101251
18	BioLegend	MHC II-PE-Cy7	116419
19	BioLegend	Ly6C-BV785	128041
20	BioLegend	PDL1-APC	124311

21	BioLegend	CD45.2-AlexaFlour700	109821
22	BioLegend	MHC II-FITC	107605
23	BioLegend	F4/80-PerCP/Cy5.5	123127
24	BioLegend	CX3CR1-PE	149005
25	BioLegend	CD80-PE/Cy5	104711
26	BioLegend	PD1-PE/Cy7	109109
27	BioLegend	CD4-BV605	100451
28	BioLegend	CD8-BV650	100741
29	BioLegend	CD25- PE/Cy5	102010
30	Ebioscience	CD4-APC-eFluor 780	47-0041-82
31	Ebioscience	CD206-APC	17-2061-80
32	Ebioscience	F4/80-PE	12-4801-82
33	ThermoFisher Scientific	Aqua Live / Dead Fixable 405	L34966
34	ThermoFisher Scientific	CD11c-APCeFluor780	47-0032-82
35	ThermoFisher Scientific	CD19	47-0193-82
36	ThermoFisher Scientific	NK1.1-APCeFluor780	47-5941-82
37	ThermoFisher Scientific	Ki67-FITC	11-5698-82
38	ThermoFisher Scientific	CD39-PerCP-eFluor710	46-0391-82
39	ThermoFisher Scientific	GranzymeB-PE	12-8898-82
40	Cell Signaling Technology	b-Actin	4970
41	Cell Signaling Technology	ATG7	8558

42	Cell Signaling Technology	Caspase 7	8438
43	Cell Signaling Technology	Caspase 9	20750
44	Cell Signaling Technology	cGAS	31659
45	Cell Signaling Technology	STING	50494
46	Cell Signaling Technology	p-TBK1	5483
47	Cell Signaling Technology	Total TBK1	3504
48	Cell Signaling Technology	ULK1	8054
49	Cell Signaling Technology	PIK3C3	4263
50	Cell Signaling Technology	ATG7	8558
51	Thermofisher Scientific	PPT1	PA5-86510
52	Spectrum chemicals	HCQ	H1126
53	Peprotech	IL-4	214-14
54	Peprotech	IL-10	210-13

55	Peprotech	IFN-γ	315-05
56	Peprotech	M-CSF	14-8983-80
57	Sigma aldrich	LPS	L-5668
58	Sigma aldrich	PPT1 siRNA pool	EMU085041
59	Origene	PPT1 siRNA single duplex	SR409088
60	Miltenyi Biotech	Tumor Digestion kit	130-096-730
61	Miltenyi Biotech	CD11b	101251
62	Santa Cruz Biotech.	ULK1 siRNA	sc-44849
63	Santa Cruz Biotech.	PIK3C3 siRNA	sc-62803
64	Santa Cruz Biotech.	ATG7 siRNA	sc-41448
65	Santa Cruz Biotech.	PPT1 siRNA	sc-142398
66	Jackson laboratory	C57BL6	Stock# 000664
67	Jackson laboratory	B6.Cg-*Braf*^{tm1Mmcm} *Pten*^{tm1Hwu} Tg(Tyr-cre/ERT2)13Bos/BosJ	Stock#013590
